# Serum Metabolomic Profiling Identifies Characterization of Non-Obstructive Azoospermic Men

**DOI:** 10.3390/ijms18020238

**Published:** 2017-01-25

**Authors:** Zhe Zhang, Yingwei Zhang, Changjie Liu, Mingming Zhao, Yuzhuo Yang, Han Wu, Hongliang Zhang, Haocheng Lin, Lemin Zheng, Hui Jiang

**Affiliations:** 1 Department of Urology, Peking University Third Hospital, Beijing 100191, China; zhezhang@bjmu.edu.cn (Z.Z.); yangyzh1987@sina.com (Y.Y.); dradam@foxmail.com (H.W.); haochenglin292@163.com (H.L.); 2 Department of Nephrology, General Hospital of Jinan Military, Jinan 250000, China; sanyuannanke1@163.com; 3 The Institute of Cardiovascular Sciences, the Institute of Systems Biomedicine, School of Basic Medical Sciences, and Key Laboratory of Molecular Cardiovascular Sciences of Ministry of Education, Peking University Health Science Center, Beijing 100191, China; liuchangjie@bjmu.edu.cn (C.L.); pkuzhao@163.com (M.Z.); 4 Department of Human Sperm Bank, Peking University Third Hospital, Beijing 100191, China; zhanghongliang@bjmu.edu.cn

**Keywords:** non-obstructive azoospermia, metabolomic, serum, HPLC-MS/MS, biomarkers

## Abstract

Male infertility is considered a common health problem, and non-obstructive azoospermia with unclear pathogenesis is one of the most challenging tasks for clinicians. The objective of this study was to investigate the differential serum metabolic pattern in non-obstructive azoospermic men and to determine potential biomarkers related to spermatogenic dysfunction. Serum samples from patients with non-obstructive azoospermia (*n* = 22) and healthy controls (*n* = 31) were examined using high-performance liquid chromatography-tandem mass spectrometry (HPLC-MS/MS). Serum metabolomic profiling could differentiate non-obstructive azoospermic patients from healthy control subjects. A total of 24 metabolites were screened and identified as potential markers, many of which are involved in energy production, oxidative stress and cell apoptosis in spermatogenesis. Moreover, the results showed that various metabolic pathways, including d-glutamine and d-glutamate metabolism, taurine and hypotaurine metabolism, pyruvate metabolism, the citrate cycle and alanine, aspartate and glutamate metabolism, were disrupted in patients with non-obstructive azoospermia. Our results indicated that the serum metabolic disorders may contribute to the etiology of non-obstructive azoospermia. This study suggested that serum metabolomics could identify unique metabolic patterns of non-obstructive azoospermia and provide novel insights into the pathogenesis underlying male infertility.

## 1. Introduction

Infertility has become a major health problem, affecting 8%–15% of reproductive-age couples worldwide, and half of these infertility cases are due to male factors [1]. Azoospermia is the most serious type of male infertility and is mainly classified into obstructive azoospermia (OA) and non-obstructive azoospermia (NOA) [2]. Most OA is caused by epydidimal duct obstruction or congenital bilateral absence of the vas deferens and exhibits normal spermatogenesis. Conversely, NOA generally results from idiopathic spermatogenic dysfunction of unknown etiology. NOA is the most challenging task for clinicians because even patients with severe oligospermia, asthenospermia, or OA could obtain a satisfactory outcome by assisted reproductive technology (ART), while patients with NOA rarely can father their own genetic offspring. Moreover, the pathogenesis of NOA is still unclear. Therefore, it is imperative to investigate the molecular mechanism underlying NOA and seek a potential treatment.

The routine evaluations to diagnose male infertility include physical examination, semen analysis, endocrine detection and other additional tests such as sperm DNA fragmentation and genetic screening [1]. Although there is an improvement in diagnostic methods, these methods still cannot satisfy the demand of clinical diagnosis, and a substantial proportion of male infertility patients cannot discover a specific cause for their defect. With the development of systems biology, approaches have been applied to address the complexity underlying human diseases [3]. “Omics” analyses could provide novel insights at a molecular level into physiological and pathological statuses, including genomic, proteomic, to the metabolomic analyses, in a comprehensive biology fashion [4]. Recently, genomic and proteomic studies have revealed several potential etiologies and identified some potential biomarkers of male infertility. Metabolomics is an approach to detect a broad range of small molecules (metabolites) in biological samples and provides a global biological fingerprint of metabolic signatures linking genetic and environmental factors within living organisms [5]. It may provide advantages that genomic and proteomic approaches do not have; thus, the assessment of metabolomics represents not only the end-product of gene/protein expression, but also the ultimate cellular signaling events resulting from interaction between genome/proteome and environmental stimuli and is closer to the actual phenotype.

Recently, interest has increased in the metabolomics approach to characterize and decipher male infertility [6]. Oligozoospermic infertile men showed altered urinary metabolic profiles compared with the fertile control population, and this differentiation indicates that oligozoospermia may have resulted from energy and antioxidative perturbations in spermatogenesis [7]. Serum metabolic analysis revealed different metabolic activity patterns in a group of uncontrolled subjects with different sperm concentrations and identified protein complement C3f as a potential biomarker [5]. Further, the metabolomics approach was used to detect testicular tissue of NOA, and the results revealed that phosphocholine concentrations were significantly lower in Sertoli-cell-only testes compared with normal cases [8]. These studies indicate the potential of the metabolomics approach applied to the investigation of male infertility.

The serum metabolome can represent global functional changes of living organism, and serum metabolites were used to investigate etiologies and predict pathological states in numerous complex disorders [9]. Previous study has demonstrated that serum metabolic changes are associated with oligozoospermic male infertility [5]. However, serum metabolomic profiles have not been well characterized in azoospermia to date. It is acknowledged that high-performance liquid chromatography-tandem mass spectrometry (HPLC-MS/MS) provides characteristics of accuracy, stability and sensitivity in metabolomics [10]. In the present study, we examined the metabolomic profiles of serum samples from patients with NOA and fertile controls with a novel approach of HPLC-MS/MS technology. Our study found unique serum metabolic characteristics in NOA compared with normal controls, which suggests that the disruption of metabolism may contribute to the etiologies of NOA. Further studies with larger sample scale and more strict criteria to examine the metabolic status in azoospermia are warranted.

## 2. Results

### 2.1. Demographics of Study Participants

A total of 22 NOA patients and 31 normal control subjects were enrolled in this study. There was no significant difference in terms of age between the infertile group and healthy controls. In the group of infertile cases, patients with azoospermia were identified on biopsy as having maturation arrest (MA) or a Sertoli-cell only (SCO) histological state. The clinical features are shown in Table 1.

### 2.2. Multivariate Statistical Analysis of Metabolic Profiles

**Figure 1:**
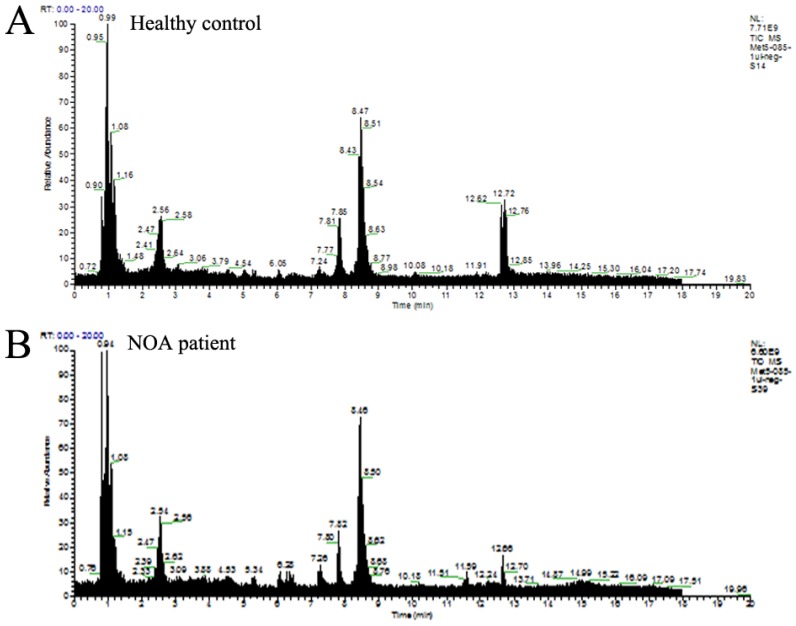
Representative base peak intensity chromatographic profiles of healthy control subject (**A**); and non-obstructive azoospermic patients (**B**).

**Figure 2:**
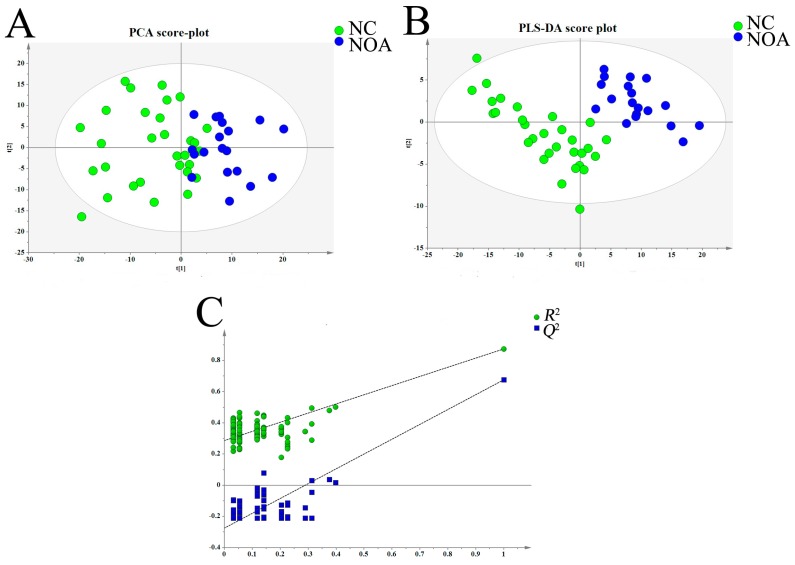
Multivariate statistical analysis of serum metabolic profiling in patients with non-obstructive azoospermia (NOA) and normal controls (NC). (**A**) principal component analysis (PCA) score plot; (**B**) partial least squares-discriminant analysis (PLS-DA) score plot; (**C**) statistical validation of established PLS-DA model with permutation analysis (100 random permutations).

The typical base peak chromatographic profiles showed a visible difference between NOA and normal controls, and spectral peaks were labeled with their corresponding metabolites (Figure 1). To compare the metabolomes between azoospermia infertile and control groups, multivariate statistical analysis was performed. In the preliminary analysis of these data, the unsupervised principal component analysis (PCA) was employed to explore clustering in the different groups. As shown in Figure 2A, although there was a small overlapping region between the azoospermic group and control cases, a trend of separation was observed in the two groups (*R*^2^(X) = 0.588, *Q*^2^ = 0.115, 4 components). Subsequently, the entire dataset was identified by a further supervised analysis of partial least squares-discriminant analysis (PLS-DA). The mode improved the clustering segregation between the two groups, and a distinct separation of azoospermia from the fertile cases was obtained (Figure 2B), and the performance parameters were as follows: *R*^2^(X) = 0.277, *R*^2^(Y) = 0.874, *Q*^2^ = 0.676, two components. The permutation test (100 random permutations) also revealed the PLS-DA method was reliable: the calculated *R*^2^ (explained variance) and *Q*^2^ (predictive ability of the model) values were lower than the original ones in the validation plot, and the regression line of *Q*^2^ intersected with the ordinate axis was below zero (intercepts: *R*^2^ = (0.0, 0.287); *Q*^2^ = (0.0, −0.275)); and no overfitting data was detected (Figure 2C).

**Figure 3:**
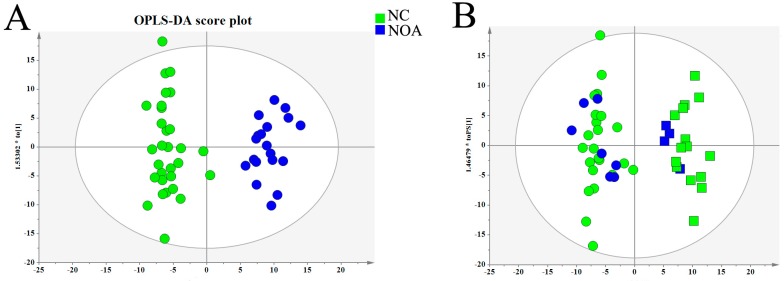
Orthogonal partial least squares-discriminant analysis (OPLS-DA) of serum metabolites in NOA and NC. (**A**) OPLS-DA score plot; (**B**) *t*-predicted scatter plot of the test set. Green squares: training set of azoospermia; green circles: training set of healthy control; blue squares: test set of azoospermia; blue circles: test set of healthy control.

As shown in Figure 3A, an even clearer discriminative profile was observed in orthogonal partial least squares-discriminant analysis (OPLS-DA) score plot (*R*^2^(X) = 0.466, *R*^2^(Y) = 0.931, *Q*^2^ = 0.797, four components, *p* value of coefficient of variationanalysis of variance (CV ANOVA) = 1.28 × 10^−11^), implying that the serum metabolic disruption was significant in the patients with NOA. However, there is no distinct segregation in the patients with different histology of MA and SCO. In addition, the 80% samples were used to build a training set of OPLS-DA, and the 20% samples remaining (test set) were subjected to the model. In addition, we found that all samples were correctly predicted in both the azoospermic infertile group and the healthy group (Figure 3B).

### 2.3. Identification of Potential Biomarkers in NOA

The distinct differentiation of azoospermia and healthy controls led us to identify potential biomarkers of metabolites that contributed to the metabolomic diversity in the two groups. First, variables (*n* = 36) with variable importance in the project (VIP) score >1 were introduced into the superset of biomarkers and used for subsequent analysis. Then, the number of candidate biomarkers was decreased to 24 after qualifying condition of the jack-knifing confidence interval >0 (Figure S1). In addition, Student’s *t*-test was performed to test the statistical significance of these differential variables. Moreover, potential biomarkers were screened by the S-plot of the established OPLS-DA model, which provides visual prediction of principal component load information. The contribution of metabolites to the class discrimination was determined by the distance from the biomarkers to the center of the S-plot. Variables with absolute *p* > 0.05 and *p*(corr) > 0.3 were selected as potential biomarkers with large contributions (Figure S2). Finally, a total of 24 metabolites were identified and listed in Table 2.

### 2.4. Metabolic Pathway and Linkages Analysis of Metabolites in NOA

To determine the possible metabolic pathway that was disrupted in NOA, all the differential metabolites were analyzed by Metabolomics Pathway Analysis (MetPA), which is based on the Kyoto Encyclopedia of Genes and Genomes (KEGG) Pathway Database. The impact value of metabolic pathway was computed by pathway enrichment and topology analysis. All disrupted metabolic pathway involved in azoospermia were listed in Table S1, and the top five potential pathways were d-glutamine and d-glutamate metabolism, taurine and hypotaurine metabolism, pyruvate metabolism, the citrate cycle (TCA cycle) and alanine, aspartate and glutamate metabolism.

**Figure 4:**
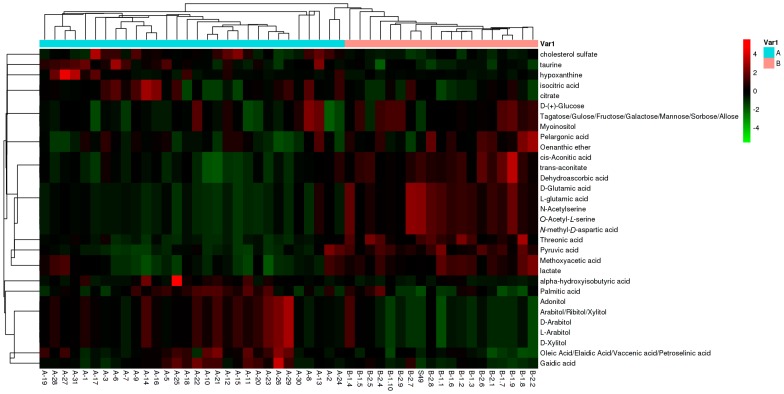
Hierarchical cluster analysis heat map of differential serum metabolites between NOA and healthy controls. Red indicates up-regulation, and green indicates down-regulation. The columns and rows represent experimental serum samples and metabolites, respectively.

**Figure 5:**
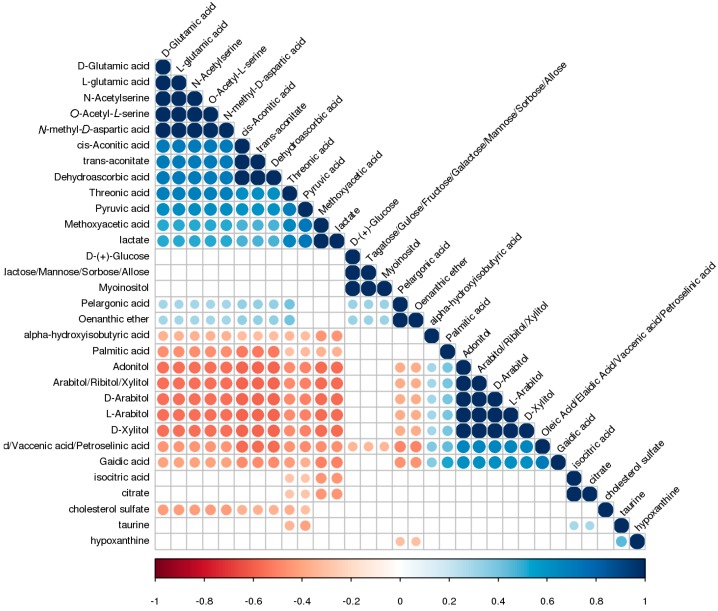
Heat map visualization of correlation analysis of differential metabolites. The colored dots indicate that the correlations between serum metabolites have statistical significance (*p* < 0.05). The red and blue dots represent positive and negative correlations, respectively.

A heat map of hierarchical clustering analysis (HCA) was performed to investigate probable discrepancies in the differential metabolite profiles between azoospermia and healthy controls. The clinical information was the major source of variance in the data, which indicated an absolute change of metabolism in the two conditions. The metabolites displayed three different clusters (Figure 4). Every cluster presented a good separation of the metabolite trend between samples fromazoospermia and healthy controls. The first cluster included cholesterol sulfate, taurine, hypoxanthine, isocitric acid, and citrate, which are involved in taurine and hypotaurine metabolism. The second cluster contained glucose, fructose myoinositol, aconitate, dehydroascorbic acid, glutamic acid, *N*-acetylserine, *N*-methyl-aspartic acid, threonic acid, pyruvic acid, methoxyacetic acid, and lactate, which are involved in glycolysis or gluconeogenesis. These compounds have higher levels in healthy controls than in patients with azoospermia. A set of highly correlated sugars, namely, adonitol, arabitol, and xylitol, were included in the last cluster metabolites, which are involved in pentose andglucuronateinterconversions. In addition, hydroxyisobutyric acid, palmitic acid, oleic acid, and gaidic acid were also included, which take part in fatty acid metabolism. Those metabolites have higher levels in azoospermia compared with healthy controls. To determine the relevance among the metabolites or the clusters, correlations in metabolites were investigated. The correlations between metabolites of the different classes were similar to the results of HCA (Figure 5). The metabolites in the second cluster are strongly correlated with each other (*r* values 0.48–0.73) and correlated with metabolites in the third cluster inversely (*r* values −0.59–−0.25).

## 3. Discussion

In the present study, we attempted, for the first time, to investigate whether serum metabolomic patterns can differentiate azoospermic patients from healthy controls with a novel approach of high-performance liquid chromatography-tandem mass spectrometry (HPLC-MS/MS) technology. Our results found a unique serum metabolomic signature in NOA compared with control subjects, and a total of 24 potential biomarkers were identified, many of which are associated with energy production, oxidative stress and cell apoptosis in spermatogenesis. Moreover, various metabolic pathways involved in glycometabolism, lipid metabolism and amino acid metabolism were disrupted in the patients with NOA.

Metabolic regulation and energy support are essential for the normal process of spermatogenesis, yet such homeostasis may be disrupted in NOA [11,12]. Our results showed a perturbation of the citrate cycle in the patients with NOA, where the concentrations of both citrate and isocitric acid are significantly decreased in the infertile cases compared with normal controls, while the amounts of lactate and pyruvic acid were increased. Similar to what we have observed, metabolomic analysis of urinary sample also revealed disrupted citrate cycles in normozoospermic infertile men [13]. Hamamah et al. examined the levels of citrate, lactate, glycerylphosphorylethanolamine (GPE) and glycerylphosphorylcholine (GPC) in human seminal plasma by ^1^H nuclear magnetic resonance, and they found that the concentrations of citrate, lactate and GPC are lower in patients with azoospermia than in healthy controls [14]. Single nucleotide polymorphisms of two genes encoding key enzymes in thecitrate cycle, namely, the succinate dehydrogenase subunits and citrate synthase gene, may be associated with impaired spermatogenesis [15]. We hypothesize that disorder of enzymes related to the citrate cycle may be a potential cause of male infertility. The concentration of palmitic acid was found to be decreased in the serum of azoospermic patients compared with healthy subjects. Palmitic acid is one of the most abundant saturated fatty acids in spermatozoa [16]. Andersen et al. reported that there is a positive correlation between the level of palmitic acid in spermatozoa and total sperm count, suggesting its important role in the productionof sperm [17]. These findings indicate that a disorder of palmitic acid contributes to impaired spermatogenesis and may be a potential target for azoospermia treatment. However, the regulation of energy production involves numerous metabolic pathways and its biological interpretation remains tricky, so further investigation is needed.

Oxidative stress, as a result of an imbalance between reactive oxygen species (ROS) and antioxidants, has long been considered a possible cause of male infertility [18,19]. Although normal levels of ROS are required for germ cell proliferation, meiosis and maturation, excessive ROS can deteriorate physiological processes and cause male infertility. Current studies have found increased levels of ROS in 30%–80% of infertile men [20,21]. Agarwal et al. reported that ROS values in semen are potential biomarkers in diagnosing male factors with a specificity of 68.8% and a sensitivity of 93.8% [22]. Our metabolomic profiles indicated that several metabolites involved in antioxidative actions decreased in NOA compared with fertile cases. Taurine, also named 2-aminoethanesulfonic acid, is the most abundant free amino acid in many tissues. As an effective free radical scavenger, taurine acts as an antioxidant primarily through both reduction of superoxide production by the electron transport chain and reversal of decreased antioxidant enzyme activity caused by ROS during spermatogenesis [23]. It has been demonstrated that taurine supplement could elevate testicular antioxidation, improve sperm quality, and increase the levels of luteinizing hormone (LH) and testosterone in physiological and pathological conditions [24]. In addition, taurine signaling initiates meiotic activity of germ cells by up-regulation of Spo11a expression in spermatogenesis [25]. These findings suggest that oxidative stress may contribute to impaired spermatogenesis and may be present in the pathogenesis of azoospermia. In addition, we found that the serum methoxyacetic acid level was significantly increased in azoospermia compared with normal controls. Methoxyacetic acid is a metabolite of ethylene glycolmonomethoxy ether in vivo and exhibits obvious reproductive toxicity [26,27]. It is reported that methoxyacetic acid induces spermatocyte apoptosis by increasing acetylation of core histones and protein kinase activity in testis germ cells. We speculate that an excessive concentration of methoxyacetic acid may be one of the reasons for spermatogenic dysfunction in azoospermic patients.

Besides acting as crucial nutrients in the energy metabolism, amino acids and other metabolites are important signaling molecules in the regulation of complex physiological process during spermatogenesis [28]. Alterations of several amino acid metabolisms and sulfur metabolisms were observed in the metabolomic profiles of NOA and normal subjects. Glutamate is an abundant free amino acid in cellular metabolism and is involved in the synthesis of other amino acids, proteins and nucleotides [29]. Here, we found that the concentration of glutamic acid is increased in infertile serum samples. Similarly, Zhang and his colleagues reported that the level of glutamine and glutamate were also elevated in asthenozoospermia compared with healthy controls [30]. However, the specific mechanism underlying disorders of glutamate metabolism is unclear and must be further investigated. Sulfonated metabolites provide substantial compounds for many endogenous molecules such as hormones and neurotransmitters, and cholesterol sulfate is the most important sterol sulfate in human plasma [31]. In the male reproductive tract, cholesterol sulfate maintains arelatively high concentration and plays a crucial role in sperm maturation [32]. Our results showed that cholesterol sulfate is significantly decreased in the group of azoospermia. It has been found that hydrolysis of cholesterol sulfate could result in sperm membrane destabilization; therefore, dysregulated cholesterol sulfonation may be present in the pathogenesis of azoospermia.

Spermatogenesis is a complicated and orchestrated process of germ cells’ self-renewal and differentiation, from spermatogonia developing into haploid spermatozoa through meiosis, and both genetic and environmental alterations can affect this process. There is increasing evidence that shows that energy metabolism and reproductive function are intimately related and metabolic regulation is important for spermatogenesis [33]. Germ cells require an adequate amount of energy substrates and nutritional support, such as carbohydrates, amino acids, lipids and vitamins; otherwise, their proliferation, differentiation and survival will suffer [12]. Studies have shown that in men, brief periods of fasting and extreme exercise reduce the level of LH and testosterone by suppression of the reproductive axis and therefore alter male reproductive function [34]. In addition, metabolism-related hormones play critical roles in the interaction between metabolism and reproduction. Thyroid hormones stimulate testicular amino accumulation, and enhance glucose transport and gamma-glutamyltranspeptidase production in Sertoli cells; leptin-deficient mice exhibit impaired spermatogenesis, accompanied byincreased germ cell apoptosis, and alteration of proapoptotic genes expression within the testis [35,36]. Metformin improves insulin resistance in patients with metabolic syndrome, accompanied by a significant increase of serum androgen levels and sperm levels and activity, which also indicates that metabolic homeostasis is crucial for male reproduction [37]. Our study found that the disruption of metabolic pathways, including the citrate cycle and glutamate and taurine metabolism, at least partially contributed to the etiologies of NOA.

It is noteworthy that there are several limitations in the present study. One is that the sample size of NOA was relatively small. Although some metabolic disorders have been found in these patients with NOA, further research with a larger sample scale isrequired to verify this potential pathogenesis. Moreover, the inter-individual metabolic variability of subjects was not well controlled because bodily metabolic activity is susceptible to various factors including environment, lifestyles and diets. Accordingly, to eliminate the influence of these confounding factors, studies with more strict criteria of subject selection and controlled animal model are needed. In the study with untargeted investigation, although some metabolites, such as lactate, taurine, and cholesterol sulfate, were confirmed by comparing their raw MS/MS with the available standards, some isomers cannot be differentiated due to the same retention time. Further studies with multiple reaction monitoring (MRM) methods to detect the interesting metabolites in azoospermia are warranted.Finally, the metabolomics profiles present only the downstream alterations of this disease, and thus, it is better to continue to study integrated genomics and proteomics to obtain a comprehensive understanding of etiology and the molecular mechanism of male infertility.

## 4. Materials and Methods

### 4.1. Study Design and Participant Recruitment

The study was approved by the Ethical Committee of Peking University Third Hospital (PUTH, 2015-0065) and conducted according to the Helsinki Declaration. The patients with NOA were enrolled from the Reproductive Center of PUTH, and healthy controls were from the Human Sperm Bank of PUTH. Every subject was fully informed of the purpose of the study and provided informed consent before the research. All subjects are ethnically Han Chinese. NOA is defined as no sperm detected after three examinations without obstruction in the reproductive tract. To eliminate the influence of other confounders that may affect fecundity and metabolism, the exclusive criterion are presented as follows: (1) subjects with malformation, trauma, tumor or infection in the reproductive system; (2) subjects with history of varicocele, cryptorchidism, orchitis, epididymitis, vas deferens or ejaculatory duct obstruction; (3) subjects with chromosome abnormality or Y chromosome microdeletions; and (4) subjects with metabolic disorders such as diabetes and hepatic disease, occupational exposure to the agents, and other known factors related to male infertility and metabolism. The healthy subjects recruited from the Human Sperm Bank of PUTH had normal physical and reproductive functionin accordance with the World Health Organization (WHO) fifth edition sperm parameters:sperm volume ≥1.5 mL, sperm concentration ≥15 × 10^6^/mL, progressive sperm ≥32%, and normal morphology ≥4%.

### 4.2. Serum Collection and Preparation

The blood samples from both NOA and healthy controls were centrifuged at 2000× *g* for 10 min, and then the obtained serum samples were stored at −80 °C immediately. Fifty-microliter serum samples were aliquoted to a 1.5-mL Eppendorf tube and mixed with 200 µL of methanol. Protein in the samples was precipitated by vortexing for 1 min and then incubated at −80 °C for 8 h, and then the supernatant was recovered following centrifugation at 14,000× *g* at 4 °C for 10 min. The supernatant was dried using a SpeedVac (Thermo Fisher, San Jose, CA, USA) with no heat.

### 4.3. HPLC-MS/MS Analysis

Untargeted metabolites screening wasperformed on Q ExactiveOrbitrap mass spectrometer (MS) with HPLC according to the calibrated manufacturer’s guidelines. A Bridged Ethylene Hybrid (BEH) Amide column was used in negative mode in LC. Mobile phase A was prepared with 10 mM ammonium acetate in 95% acetonitrile. The pH was adjusted to 9.0 using an ammonium hydroxide solution. Mobile phase B was prepared with 10mM ammonium acetate in 50% acetonitrile, and the pH was adjusted to 9.0 with ammonium hydroxide solution. The column temperature was 35 °C. The elution solution was 5% B (A:B; 95:5, by volume) for 2 min followed by a linear gradient up to 45% B for the next 6 min, up to 85% for the next 10 min, and up to 95% for the next 1 min, where it was held for 2 min before it was returned to 5% B for 2.1 min. The flow rate was 250 μL/min of phase B.

The HPLC system was coupled to a Q ExactiveOrbitrap mass spectrometer (Thermo Fisher, San Jose, CA, USA) equipped with a heated electrospray ionization (HESI) probe. The spray voltage was set to 2.5 kV, whereas the capillary temperature was held at 320 °C. The sheath gas flow was set to 35 units and the auxiliary gas set to 10 units. These conditions were held at negative ionization mode acquisitions. Resolution of 70,000 and 17,500 was used in MS and MS/MS acquisition respectively. One precursor scanned followed by 10 MS/MS spectra were performed; 30% + 50% of normalized collision dissociation was applied in the experiment. The same LC conditions and buffers were used for all MS experiments, and the scan range was between *m*/*z* 80 and 1200. The duty cycle(s) is 1.2 s. External mass calibration was performed before experiment. Serum samples were randomized in the sequence and metabolites were profiled by single injection including with 53 pool samples, 3 blanks, and 5 quality control (QC) samples throughout the analysis. The coefficient of variation (CV) of metabolites for the same QC samples were less than 10%.

### 4.4. Data Processing and Statistical Analysis

The obtained raw spectrogram was processed using Tracefinder 3.2 (Thermo Fisher Scientific). Metabolites have two levels of identification, one with accurate mass matching and the other with MS/MS confirmation. Mass tolerance for database search is 8 ppm for MS, and 15 ppm for MS/MS. The instrument stability was monitored using QC samples. The intensity of extracted variables was normalized to the total areas to reduce the variations from sample injection and enrichment factor. After peak deconvolution, alignment, integration, and normalization, a table containing retention times, exact mass pairs, and normalized intensities of each variable were obtained for multivariate statistical analysis. Then, all normalized Pareto-scaled data were imported into SIMCA-P v13.0 software (Umetrics AB, Umea, Sweden) for multivariate statistical analysis. First, PCA, an unsupervised pattern recognition approach, was employed to diminish the dimensionality of the variable and observe any intrinsic clusters between azoospermia and control groups. Then, PLS-DA and OPLS-DA were performed to remove irrelevant variability and to obtaina better class separation and to identify potential biomarkers in a supervised manner. Compared with the model of PLS-DA, OPLS-DA maximizes the variation between the specified groups and minimizes the variation between the individual replicates, and yields enhanced the interpretability of the multivariate model, and did not affect the predictive capability. The number of components of PCA, PLS-DA and OPLS-DA were 4, 2 and 4, respectively. To avoid examination of the goodness of fit in the PLS-DA model, random permutation test and internal cross validation were performed.

Potential biomarkers were identified according to the significance of their contribution to variable classification, which was determined by the VIP plot and jack-knifing confidence interval (≥0) in the OPLS-DA model. Then, the discriminant metabolites were further validated using two-tailed Student’s *t*-test (*p* < 0.05) in Statistical Product and Service Solutions (SPSS) 20.0 software. Subsequently, the potential biomarkers obtained were identified as described previously. Briefly, the spectral feature and information of these metabolites were required by MS and MS/MS analysis of raw serum metabolomics data, and the obtained raw spectrograms were processed using Tracefinder 3.2. The precursor and fragment information of metabolites were imported into Tracefinder, and MS/MS mass spectral library search was performed to evaluate the similarity between the MS/MS spectrum of samples and the standard spectrum of the MS/MS mass spectral library, and the potential biomarkers of metabolites were selected according to mass library scores. All potential biomarkers were confirmed by matching METLIN database (Available online: http://metlin.scripps.edu), Human Metabolome Database (Available online: http://www.hmdb.ca) and MassBank (Available online: http://www.massbank.jp) in terms of accurate mass, retention time, and fragments. Biomarker identities were finally confirmed by comparison with commercial standards or online databases and literature (when standards were unavailable). The representative metabolites raw MS/MS and standard MS/MS spectrum were showed in the Figure S3. Metabolomics Pathway Analysis (MetPA) is a web-based and visual tool, which could be used to analyze metabolomic data within the biological context of metabolic pathways and identify the most relevant pathways in a metabolic study [38]. To explore the related metabolic pathways of these potential biomarkers, all differential metabolites were introduced to metaboanalyst (Available online: www.metaboanalyst.ca) and analyzed by MetPA, which is based on the KEGG Pathway Database (Available online: http://www.genome.jp/kegg), and the figure of pathway analysis with MetPA was showed as Figure S4.

Quantitative data are presented as the mean ± SD. A normality test was used to explore the data distribution, two-tailed Student’s *t*-test was used for statistical analysis with SPSS, and *p* < 0.05 was considered statistically significant. HCA was performed by R software (version 3.1.3) to visualize the differentiated metabolites profiles. The data were normalized based on the abundance of the internal standard and transformed with unit variance scaling. R-language was also used to perform a Spearman correlation test of correlative analysis between potential biomarkers. The false discovery rate (FDR) significance criterion (*p* < 0.05) was used to avoid false-positive results.

## 5. Conclusions

In this study, we examined the metabolomics characterization of NOA using HPLC-MS/MS to evaluate their differentiations in terms of metabolites and metabolic pathways. Metabolic patterns of serum samples from infertile and fertile men were demonstrated to be markedly different and reflected the complex networks of metabolic alteration in patients with NOA. Several potential metabolites were identified that are closely associated with energy production, oxidative stress and cell apoptosis in spermatogenesis. Biosynthesis and metabolism of these metabolites may contribute to the etiologies of azoospermia. Our findings suggested that serum metabolomics fingerprinting could provide a promising screening approach to identify unique metabolic patterns of NOA, and eventually aid etiological diagnosis and therapy intervention. Further studies with larger samples and more strict criteria are required to reveal the pathological mechanism underlying male infertility.

## Figures and Tables

**Table 1:** Clinical features and biopsy results of azoospermic patients and normal subjects.

Clinical Features	NC	NOA
Total number	31	22
Age (Years, Mean ± SD)	28.1 ± 4.5	30.6 ± 4.3
Testosterone < 14 nmol/L	NA	2
FSH > 11.1 mIU/mL	NA	9
Biopsy findings	NA	
Maturation arrest		12
Sertoli-cell only		10
Cytology findings	NA	
Spermatids or sperm		5

NA, not applicable; FSH, follicle stimulating hormone; NC: normal control; NOA: non-obstructive azoospermia.

**Table 2:** Potential serum biomarkers of non-obstructiveazoospermia.

Compound Name	Formula	KEGG ID	Mass ∆ (Da)	VIP	Fold Change	Retention Time (min)	Mass Error (ppm)	Mass Library Score
Oleic acid	C_18_H_34_O_2_	C00712	282.2559	6.33	0.68	0.9891	0	30
Lactate/Methoxyacetic acid	C_3_H_6_O_3_	C01432/NA	90.0317	5.87	1.43	7.2385	6	83
Citrate/isocitric acid	C_6_H_8_O_7_	C00158/C00311	192.0270	3.17	0.62	12.9001	0	100
Palmitic acid	C_16_H_32_O_2_	C00249	256.2402	3.16	0.79	0.9891	0	NA
*O*-Acetyl-l-serine/*N*-methyl-d-aspartic acid/*N*-Acetylserine/l(d)-glutamic acid	C_5_H_9_NO_4_	C00979/C12269/NA/C00025	147.0532	2.10	2.32	11.1536	1	70
Trans(*cis*)-aconitate/Dehydroascorbicacid	C_6_H_6_O_6_	C02341/C05422	174.0164	1.97	1.47	11.7449	0	83
Pyruvic acid	C_3_H_4_O_3_	C00022	88.0160	1.81	2.00	4.9982	6	67
Tagatose/Gulose/Fructose/d-(+)-Glucose	C_6_H_12_O_6_	C00795/C00267	180.0634	1.81	1.13	8.4792	0	97
Threonic acid	C_4_H_8_O_5_	NA	136.0372	1.36	2.05	9.2863	0	66
Gaidic acid	C_16_H_30_O_2_	NA	254.2246	1.35	0.61	1.21	0	44
Taurine	C_2_H_7_NO_3_S	C00245	125.0147	1.33	0.65	8.3418	2	NA
Pelargonic acid/Oenanthic ether	C_9_H_18_O_2_	C01601/NA	158.1307	1.26	1.34	1.4672	0	31
Cholesterol sulfate	C_27_H_46_O_4_S	C18043	466.3117	1.24	0.57	0.8231	0	60
Hypoxanthine	C_5_H_4_N_4_O	C00262	136.0385	1.09	0.34	4.8046	0	69
Arabitol/Ribitol/Xylitol	C_5_H_12_O_5_	C00532	152.0685	1.07	0.63	2.8157	0	22
α-hydroxyisobutyric acid	C_4_H_8_O_3_	C01188	104.0473	1.04	0.47	7.1775	4	78

KEGG, Kyoto Encyclopedia of Genes and Genomes; NA, not applicable; fold change value refers to the “non-obstructiveazoospermia vs. control group” change values.

## Supplementary Materials

Supplementary materials can be found at www.mdpi.com/1422-0067/18/2/238/s1.

## Abbreviations

ART	Assisted Reproductive Technology
FDR	False Discovery Rate
GPC	Glycerylphosphorylcholine
GPE	Glycerylphosphorylethanolamine
HCA	Hierarchical Clustering Analysis
HPLC-MS/MS	High-Performance Liquid Chromatography-tandem Mass Spectrometry
LH	Luteinizing Hormone
MA	Maturation Arrest
NOA	Non-obstructiveAzoospermia
OA	Obstructive Azoospermia
OPLS-DA	Orthogonal Partial Least Squares-Discriminant Analysis
PCA	Principal Component Analysis
PLS-DA	Partial Least Squares-Discriminant Analysis
ROS	Reactive Oxygen Species
VIP	Variable Importance in the Project
