# Supplementary material for: Serum Metabolomic Profiling Identifies Characterization of Non-Obstructive Azoospermic Men
Source: Int J Mol Sci. 2017 Jan 25;18(2):238. doi: 10.3390/ijms18020238 (PMC5343775; doi:10.3390/ijms18020238)
Supplement: Supplementary file 1 [file ijms-18-00238-s001.pdf]

Supplementary Materials:Serum Metabolomic Profiling Identifies Characterization of Non-Obstructive Azoospermic Men

Zhe Zhang, Yingwei Zhang, Changjie Liu, Mingming Zhao, Yuzhuo Yang, Han Wu, Hongliang Zhang, Haocheng Lin, Lemin Zheng and Hui Jiang

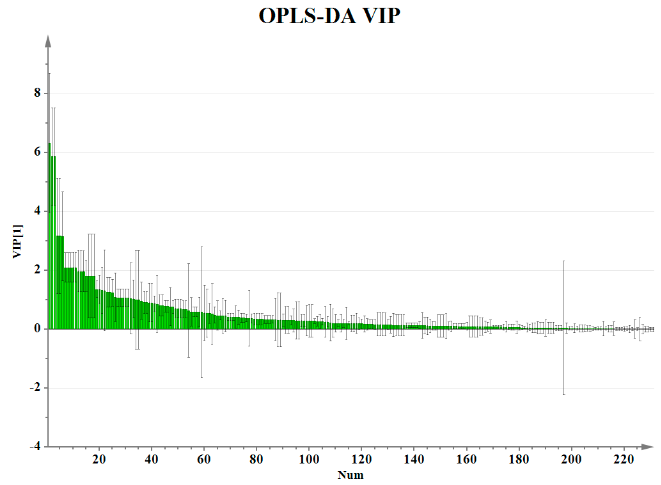

Figure S1. Variable influence on projection (VIP) of established orthogonal partial least squares-discriminant analysis (OPLS-DA) model. Variables with negative jack-knifing confidence intervals were eliminated.

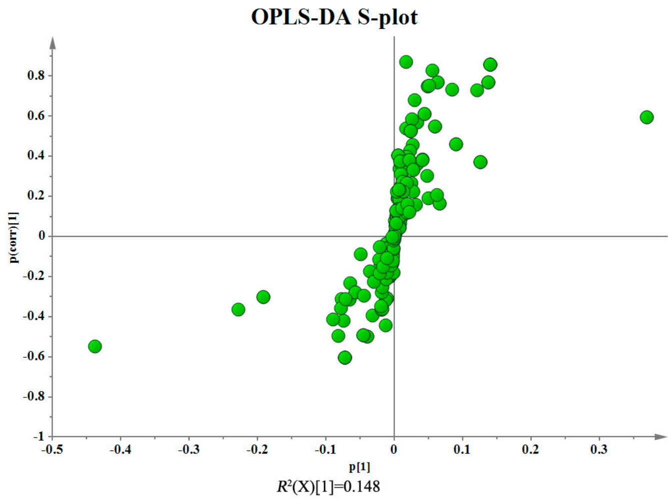

Figure S2. S-plot diagram of established OPLS-DA model. Variables that are closer to the lower-left and upper-right corners have greater contributions to group differentiation.

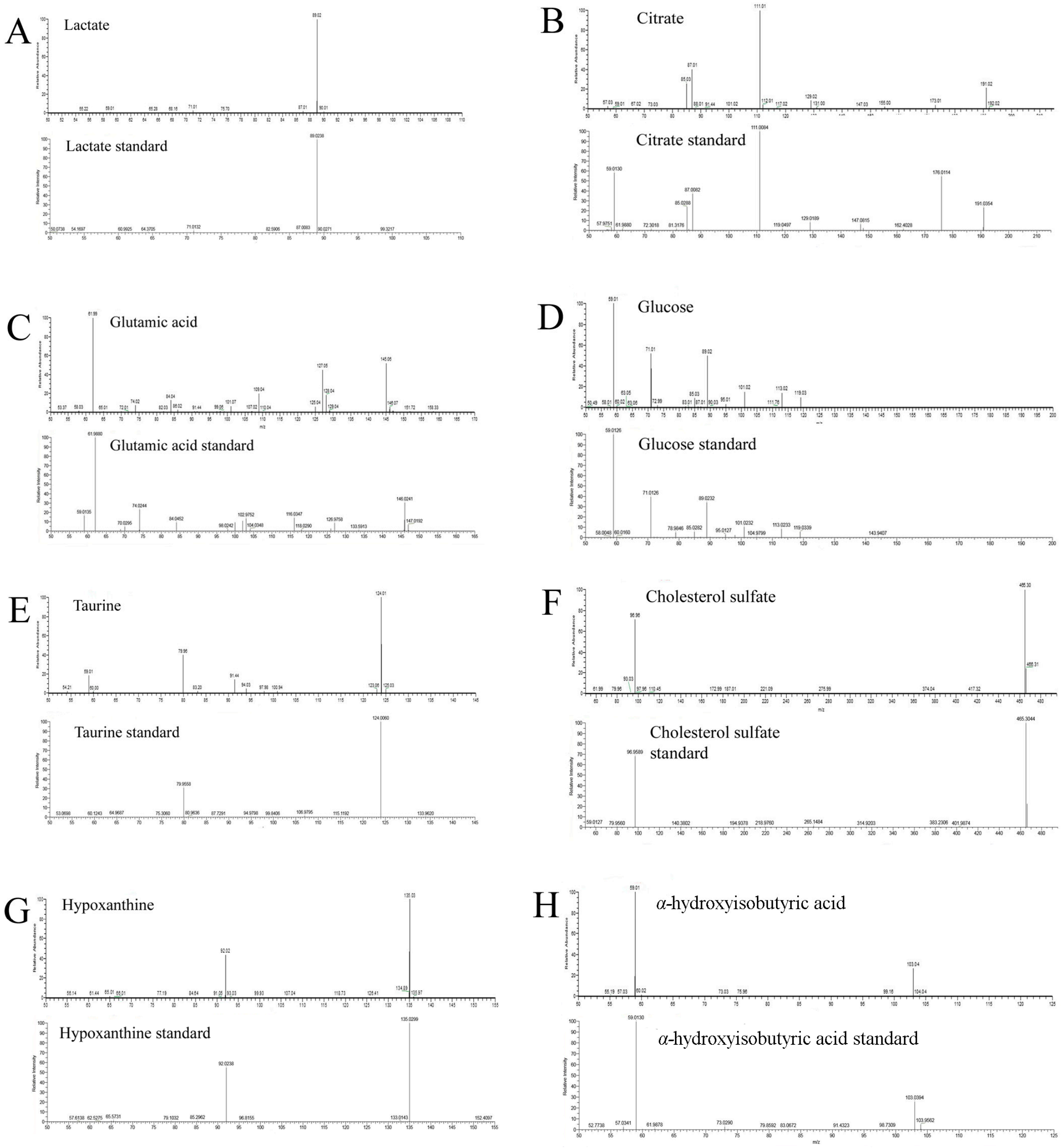

Figure S3. MS/MS spectra of lactate (**A**); citrate (**B**); glutamic acid (**C**); glucose (**D**); taurine (**E**); cholesterol sulfate (**F**); hypoxanthine (**G**); α-hydroxyisobutyric acid (**H**) in the serum of non-obstructive azoospermia with comparison to their standard.

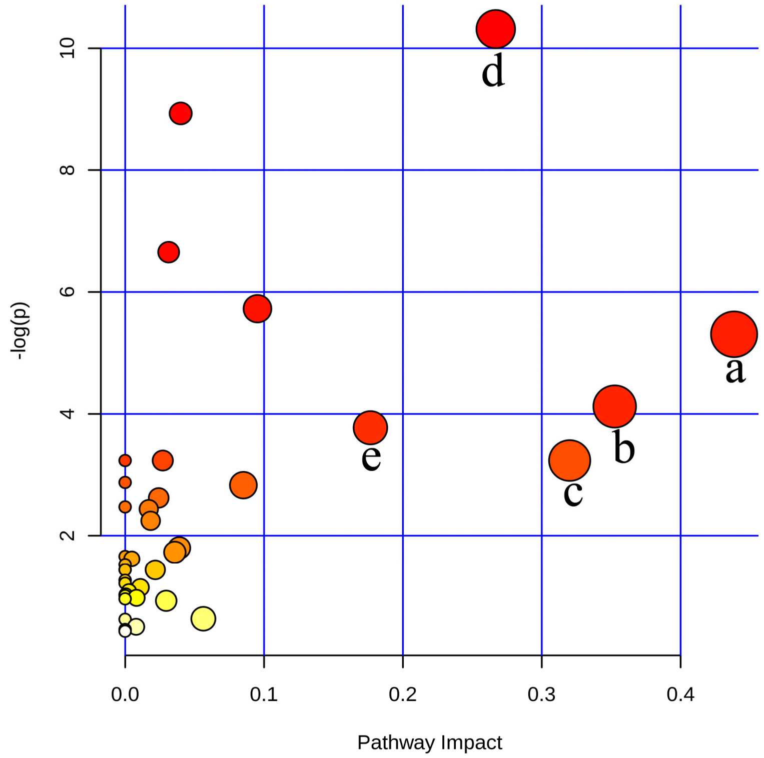

Figure S4. Metabolomics Pathway Analysis (MetPA) of disrupted serum metabolic pathway in patients with non-obstrucive azoospermia. (**a**) D-glutamine and D-glutamate metabolism; (**b**) taurine and hypotaurine metabolism; (**c**) pyruvate metabolism; (**d**) the citrate cycle and (**e**) alanine, aspartate and glutamate metabolism.

Table S1. Disrupted serum metabolic pathway in patients with non-obstrucive azoospermia.

Metabolic Pathway	Total	Expected	Hits	Raw *p*	−log (Raw *p*)	Holm Adjust	FDR	Impact
D-Glutamine and D-glutamate metabolism	11	0.10968	2	0.004962	5.3059	0.37712	0.079393	0.4385
Taurine and hypotaurine metabolism	20	0.19942	2	0.016229	4.121	1	0.21639	0.35252
Pyruvate metabolism	32	0.31907	2	0.0394	3.234	1	0.3152	0.3201
Citrate cycle (TCA cycle)	20	0.19942	4	0.000033	10.314	0.002653	0.002653	0.26688
Alanine, aspartate and glutamate metabolism	24	0.2393	2	0.02301	3.7718	1	0.26297	0.17664
Glycolysis or Gluconeogenesis	31	0.3091	3	0.003261	5.7256	0.25113	0.065228	0.0953
Butanoate metabolism	40	0.39884	2	0.059048	2.8294	1	0.39366	0.08516
Aminoacyl-tRNA biosynthesis	75	0.74782	1	0.53393	0.62748	1	1	0.05634
Pentose and glucuronateinterconversions	53	0.52846	5	0.000132	8.9306	0.01045	0.005291	0.04002
Sulfur metabolism	18	0.17948	1	0.16558	1.7983	1	0.77921	0.03898
Arginine and proline metabolism	77	0.76776	2	0.17779	1.7272	1	0.79018	0.03582
Glyoxylate and dicarboxylate metabolism	50	0.49855	4	0.00129	6.6535	0.10058	0.034387	0.03137
Fatty acid metabolism	50	0.49855	1	0.39725	0.9232	1	0.9347	0.02959
Vitamin B6 metabolism	32	0.31907	2	0.0394	3.234	1	0.3152	0.02712
Ascorbate and aldarate metabolism	45	0.44869	2	0.072732	2.621	1	0.44758	0.02419
Valine, leucine and isoleucine biosynthesis	27	0.26921	1	0.23818	1.4347	1	0.79393	0.02173
Cysteine and methionine metabolism	56	0.55837	2	0.10595	2.2448	1	0.52974	0.01838
Starch and sucrose metabolism	50	0.49855	2	0.087346	2.4379	1	0.46585	0.01703
Glutathione metabolism	38	0.37889	1	0.31871	1.1435	1	0.93282	0.01095
Primary bile acid biosynthesis	47	0.46863	1	0.37847	0.97162	1	0.93282	0.00822
Purine metabolism	92	0.91732	1	0.60933	0.49539	1	1	0.00791
Selenoamino acid metabolism	22	0.21936	1	0.19863	1.6163	1	0.79393	0.00482
Galactose metabolism	41	0.40881	1	0.33922	1.0811	1	0.93282	0.00276
Histidine metabolism	44	0.43872	1	0.35913	1.0241	1	0.93282	0.00051

FDR, False Discovery Rate.
